# Ayurveda and Epigenetics

**DOI:** 10.3390/medicina56120687

**Published:** 2020-12-11

**Authors:** Hari Sharma, Robert Keith Wallace

**Affiliations:** 1 Ohio State Integrative Medicine, Department of Family and Community Medicine, College of Medicine, The Ohio State University, Columbus, OH 43221, USA; 2 Department of Physiology and Health, Maharishi International University, Fairfield, IA 52556, USA

**Keywords:** Ayurveda, epigenetics, genotype, phenotype, Prakriti, doshas

## Abstract

Ayurveda is a comprehensive, natural health care system that originated in the ancient Vedic times of India. Epigenetics refers to the external modification of DNA that turns genes on and off, affecting gene expression. This occurs without changes in the basic structure of the DNA. This gene expression can have transgenerational effects. The major factors that cause epigenetic changes are lifestyle and behavior, diet and digestion, stress, and environmental factors. Ayurveda addresses these factors, thereby affecting the Deha (body) Prakriti (psychophysiological constitution), which corresponds to the phenotype, and indirectly the Janma (birth) Prakriti, which corresponds to the genotype. Thus, it is proposed that epigenetics is an important mechanism of Ayurveda. This correlation and understanding will lead to better communication and understanding with the current medical system, and lead to better integration of both sciences in the management of optimal health. In addition, research on Ayurvedic modalities affecting gene expression will further increase correlation and understanding between the current medical system and Ayurveda.

## 1. Introduction

Ayurveda is a comprehensive, natural system of health care that originated in the ancient Vedic times of India. Ayurveda is a Sanskrit term that translates as the “Science of Life.” Sanskrit was the main language used in communication and teaching in the ancient Vedic times. In the context of current knowledge, many of the original concepts of Veda (translates as “knowledge”) and Ayurveda are not clearly understood, and this results in varied interpretations in current communication. Additionally, many of the terms originally used cannot be properly translated in current terminology, and some terms have no corresponding counterparts in English, which results in confusion and misunderstanding. This article proposes a correlation of the ancient Ayurvedic concepts and terminology with the current understanding of cellular physiology. Topics covered include genotype and phenotype and their correlation with Janma (birth) Prakriti and Deha (body) Prakriti (psychophysiological Ayurvedic constitution), and epigenetics and its correlation as an important mechanism of Ayurveda.

The difference between living and non-living entities is the presence of deoxyribonucleic acid (DNA) in living species. In the living species, the difference between them is in the order of the bases (adenine–thymine, guanine–cytosine) in the DNA [1]. All nucleated cells contain DNA. The combination of DNA creates genes. It is estimated that humans have approximately 20,000 genes. Less than 2% of these are coding genes that express themselves and are known as the genotype. The genotype refers to the part of the genetic makeup that determines specific characteristics of an individual. It is stable and non-changing unless there is toxic damage. The genotype is responsible for the development of the phenotype of the individual [1]. The phenotype refers to the physical properties of an individual—appearance, development, and behavior. Whatever one does in one’s life—knowingly or unknowingly—affects the phenotype and changes the expression of the genotype accordingly. The four major factors affecting the phenotype are lifestyle and behavior, diet and digestion, stress, and environmental factors [1].

## 2. Genotype and Phenotype in Ayurveda

In Ayurvedic terms, the Janma Prakriti or birth Prakriti does not change and is the foundation of the psychophysiological constitution or Deha Prakriti (body Prakriti), which changes and is dynamic. The genotype corresponds to Ayurvedic birth Prakriti and the phenotype corresponds to Ayurvedic Deha Prakriti. Disturbance in the Deha Prakriti is known as Vikriti in Ayurveda, which correlates with disorders and diseases in the current medical system. Manohar has described the presence of vivid accounts in the ancient Ayurvedic texts about the inheritance of diseases and the genetic basis for the transmission of such diseases from parents to progeny [2]. The Ayurvedic understanding that Deha Prakriti has a genetic basis has been corroborated by current scientific research. For example, the phosphoglucomutase 1 (*PGM1*) gene has been correlated with Pitta Prakriti (one of the Prakriti types) [3]. Research on human leucocyte antigen (*HLA*) gene polymorphism showed a reasonable correlation between HLA type and Prakriti type [4].

**Figure 1:**
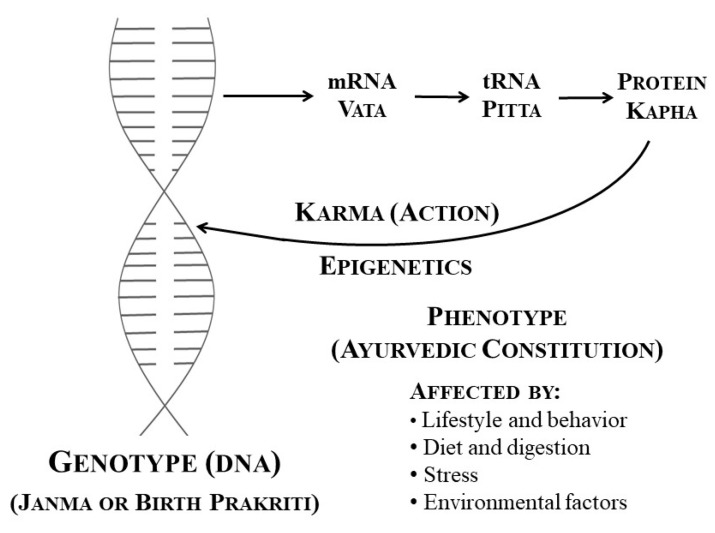
DNA and cellular function and correlation with Ayurveda. mRNA, messenger ribonucleic acid; tRNA, transfer ribonucleic acid; DNA, deoxyribonucleic acid. Modified and reprinted from [1], with permission from SelectBooks, 2018.

According to Ayurveda, there are three governing principles of the physiology, known as doshas. The three doshas are Vata, Pitta, and Kapha. Vata governs motion, flow, and communication, including the flow of the blood, the beating of the heart, the transmission of nerve impulses, etc. Pitta regulates digestion, metabolism, and transformation. Kapha governs the structure of the body. These three doshas make up the psychophysiological constitution. In the process of DNA expression, the two strands of DNA separate and the knowledge present in the strand is replicated and comes out as messenger ribonucleic acid (mRNA). The knowledge carried in mRNA is then utilized by transfer RNA (tRNA), which lines up the designated amino acids to form the specified protein (Figure 1). It is proposed that mRNA, tRNA, and protein have features and properties that represent Vata, Pitta, and Kapha at the cellular level. Messenger RNA corresponds with Vata (transmission of information), tRNA corresponds with Pitta (transformation), and protein corresponds with Kapha (structure) [1] (Figure 1).

## 3. Epigenetics and Ayurveda

Epigenetics refers to the external modification of DNA that turns genes on and off, affecting gene expression. This occurs without changes in the DNA sequence. This process produces a change in the phenotype without a change in the genotype [1]. In brief, DNA methylation, histone modification, chromatin remodeling, and micro RNA (miRNA) are involved in modifying DNA expression. DNA methylation is a process in which methyl groups are added to the DNA molecule. This process changes the activity of the DNA [5]. Histones are proteins that DNA wraps around in the nucleus, forming chromatin. This process condenses DNA into a more compact form and protects the DNA structure and sequence. Chromatin can condense or it can relax, thereby changing the expression of DNA. Histones play a major role in the condensation and relaxation of chromatin and thereby affect DNA expression [6]. Chromatin remodeling refers to the rearrangement of chromatin from a condensed state to a transcriptionally accessible state, allowing transcription factors or other DNA-binding proteins to access DNA and control gene expression [7]. MicroRNA refers to small non-coding RNA molecules that “silence” or stop the functioning of mRNA [8].

It is estimated that 90% of life is controlled by epigenetics—the changes in gene expression brought about by what one does in one’s life. Whatever is done to the phenotype or Ayurvedic psychophysiological constitution (Deha Prakriti) is relayed back to the DNA, which changes its expression accordingly. Thus, the process of epigenetics represents action (Karma—the Sanskrit word for “action”) on the level of the cells (Figure 1). In the field of physics, this is represented by Newton’s Third Law of Motion: For every action there is an equal and opposite reaction. Another way of putting it is the Biblical saying “As you sow, so shall you reap.” Every cell is going through this process. Factors that cause epigenetic changes affect DNA expression and this can also be transmitted to the progeny [9].

As mentioned previously, there are four major factors that affect one’s life. These factors are addressed in Ayurveda for maintaining health and preventing disease. They are lifestyle and behavior, diet and digestion, stress, and environmental factors. If an individual’s actions are in the positive direction, Deha Prakriti remains balanced and health is maintained. If actions are not in the positive direction, Deha Prakriti becomes imbalanced, creating Vikriti, and disease manifests. This whole process occurs through the mechanism of epigenetics. These epigenetic changes can be reversed [10]. Ayurveda addresses the factors that cause epigenetic changes, and it thereby affects both the phenotype and expression of the genotype in a positive manner. Thus, it is proposed that epigenetics is an important mechanism of Ayurveda [1,11].

A further correlation between epigenetics and Ayurveda relates to the personalized nature of the process of epigenetics and the health care system of Ayurveda. Each individual holds their health in their own hands. Through the process of epigenetics, their actions affect their health in a direct and very personal way, which can help prevent disease or lead to disease. Ayurveda is a prevention-oriented system of health care that provides detailed recommendations that are personalized for each individual based on their unique Prakriti and Vikriti.

## 4. Research on the Four Major Factors that Cause Epigenetic Changes

A growing body of evidence demonstrates the importance of the four major factors that cause epigenetic changes in the expression of DNA [10,12]. It has been identified that diet, obesity, physical activity, tobacco smoking, alcohol consumption, environmental pollutants, psychological stress, and working the night shift might modify epigenetic patterns [13]. For example, polyphenols, which are natural compounds widely found in plant foods, have been shown to modify the activity of DNA methyltransferases, histone acetylases, and histone deacetylases, inducing reversibility of epigenetic dysregulation. Epigenetic biomarkers of obesity, including genes involved in adipogenesis, methylation patterns of obesity-related genes, and inflammation genes could help predict susceptibility and prevent obesity. Physical activity has been associated with higher methylation in peripheral blood lymphocytes of long interspersed nucleotide element-1 (LINE-1) elements, which are a class of repeated sequences that are highly repeated in the human genome. Low methylation in these elements is associated with inflammatory responses and chromosomal instability [13].

Regarding tobacco smoking, cigarette smoke condensate decreases nuclear levels of certain histone modifications in respiratory epithelial cells, with these alterations being similar to changes in histone modifications found in lung cancer tissues. With regard to alcohol consumption, a population-based case–control study showed the association between LINE-1 hypomethylation in blood leukocyte DNA and gastric cancer was stronger among individuals who were current alcohol drinkers. An occupational study examined the effects of exposure to air pollution, specifically particulate matter and metal components, on miRNA expression in workers at an electric-furnace steel plant. It was found that two miRNAs related to oxidative stress and inflammation were overexpressed and positively correlated with the levels of lead exposure and oxidative DNA damage [13].

Regarding psychological stress, research has shown hypermethylation of the glucocorticoid receptor gene in suicide victims with a history of childhood abuse, but not in controls or suicide victims who did not experience childhood abuse. Several epidemiological studies have shown that working the night shift can have negative effects on the health and well-being of workers. A study on a population of night-shift workers showed alterations in blood DNA methylation, including changes in gene-specific methylation of inflammatory genes [13]. The following is further research on the four major factors that cause epigenetic changes.

### 4.1. Lifestyle and Behavior

Epigenetic dysregulations may play an important role in the onset, progression, and pathogenesis of various human disorders and diseases, including cancer and cardiovascular, neurodegenerative, and autoimmune diseases [14]. A study on men with prostate cancer showed that a lifestyle intervention program that included meditation, breathing exercises, aerobic exercise, and a vegetarian diet changed the expression of 500 genes, including the downregulation of disease-promoting genes involved in tumor formation [15]. A study on patients with coronary artery disease showed that a lifestyle modification program that included stress management, aerobic exercise, and a vegetarian diet resulted in successful and sustained modulation of gene expression that ameliorated cardiovascular risk [16]. A study on the impact of a health promotion intervention that included increased physical activity and increased intake of fruits/vegetables impacted patterns of DNA methylation in gene regions related to immune cell metabolism, tumor suppression, and overall aging [17]. Two additional studies have shown a linkage between exercise and DNA methylation, with concordant health-enhancing changes in the phenotype [18,19].

### 4.2. Diet and Digestion

Dietary nutrients and bioactive food components are epigenetic regulators that modify gene expression. Diet can modify epigenetic mechanisms by regulating DNA methylation, histone modifications, chromatin remodeling, and changes in miRNA expression [10,20]. For example, foods that contain B vitamins can influence DNA methylation [21]. Curcumin, one of the components of *Curcuma longa* L. (turmeric), is a histone deacetylase inhibitor, as demonstrated in B cell non-Hodgkin lymphoma cells. The expression levels of several histone deacetylase enzymes were downregulated following curcumin treatment. The dysfunction of histone deacetylases is associated with the manifestation of several different types of cancer [22]. In breast cancer cells, curcumin significantly downregulated both estrogen receptor alpha (ERα) and p53 protein levels, with a concomitant decrease in breast cancer cell viability. Both ERα and p53 are known to contribute to the formation and progression of hormone-dependent breast cancer [23].

In addition to curcumin, other bioactive food components have been shown to modulate epigenetic events, with epigenetic targets that are associated with breast cancer prevention and therapy. These bioactive ingredients include dietary polyphenols, epigallocatechin gallate from green tea, genistein from soybean, isothiocyanates from plant foods, resveratrol from grapes, and sulforaphane from cruciferous vegetables [24]. These bioactive food components have shown similar results in other types of cancer [25]. In light of these findings, an “epigenetics diet” has been proposed, which would utilize these bioactive dietary compounds to neutralize epigenetic aberrations as a form of cancer treatment, and be utilized as cancer prevention [26].

In Ayurveda, optimal digestion is considered to be of prime importance in maintaining health. A nutritious diet will not have the maximum impact in promoting health unless the digestive capacity is optimal. Balanced Agni (digestive fire) is critical for proper digestion to occur. If the Agni is not functioning properly, the food will not be properly digested and this can lead to the production of Ama, a toxic byproduct of incomplete digestion that is linked to disorders and diseases. Research has found that the response to food intake and individual nutrients includes epigenetic events [27]. Bile acids, which are necessary for lipid digestion and absorption, are also signaling molecules. Bile acid synthesis is transcriptionally regulated in relation to the fasted-to-fed cycle. The underlying mechanisms include chromatin remodeling at promoters of key genes involved in their metabolism [27].

Herbs have been shown to exhibit epigenetic mechanisms [28]. A study on herbs showed that 36% interacted with histone-modifying enzymes and 56% of these promoted chromatin condensation [29]. Withaferin A, a component of *Withania somnifera (L.) Dunal* (Ashwagandha), has been shown to downregulate DNA methyltransferases and histone deacetylase in breast cancer cells and induce apoptosis of these cells [30,31].

### 4.3. Stress

Exposure to stressors can change epigenetic marks, or tags, and influence the way genes are expressed [32]. Stress-associated epigenetic changes have been correlated with depression [33]. Feelings of anger, stress, frustration, and fear cause the DNA to become shorter and tighter, and switch off many codes. In contrast, feelings of gratitude, love, and appreciation cause the DNA to relax, and the strands unwind and begin to express [34]. A pilot study on telomerase gene expression in hypertensive patients showed that stress reduction through meditation and lifestyle modifications increased telomerase gene expression [35]. In light of these research studies, stress management can be viewed as a crucial requirement for health and well-being. In this regard, Ayurveda has long included a thorough understanding of meditation as a technique for stress management and the development of consciousness.

### 4.4. Environmental Factors

Environmental factors can alter gene expression through epigenetic mechanisms [10]. Research has shown that environmental toxicants such as diesel exhaust particles, cigarette smoke, and inorganic arsenate can silence certain tumor suppressor genes, resulting in carcinogenesis [36]. Chronic sun exposure causes epigenetic changes in the skin. Hypomethylation was seen in sun-exposed epidermis samples from older individuals [37].

Epigenetic changes can affect future generations. Nutrition abnormalities, environmental toxicants, and environmental stress can promote epigenetic alterations that are transmitted to subsequent generations, resulting in disease [9]. It has been shown that exposure to air pollution during gestation affects the gestating mother, her embryo, and its developing germ line, thereby influencing the third generation’s phenotype [38]. Many effects of traumatic stress can be transmitted to future generations, even when individuals from these generations are not exposed to a traumatic stressor [39].

As mentioned previously, Ayurveda addresses the four major factors that cause epigenetic changes, for the maintenance of health and prevention of disease. As a comprehensive system of health care, there are multiple aspects of Ayurveda that relate to these four factors. For example, Dinacharya (daily routine) recommends the optimal times for getting up in the morning, eating, exercising, meditating, etc., to stay in tune with the natural rhythms of nature that support optimal health. Ratricharya (night routine) recommends the optimal time to go to bed to ensure a good night’s sleep, which is considered one of the main pillars of health in Ayurveda. It is this aspect of Ayurveda that recommends against working the night shift, due to the adverse physiological changes that can result. Ritucharya (seasonal routine) includes recommendations for each season, e.g., eating cooling foods during the summer and minimizing hot, spicy foods.

Other areas of Ayurveda that provide health-promoting recommendations include Ahara-Vihara (diet and guidelines for eating), Sadvritta (social and personal behavior), Manasa Tivra (mental stress) and Manas Vritti (mental fluctuations), and Paryavarana (environment, including home and workplace). With regard to Manas (mind), mental fluctuations are created by the incoming information received through the five senses (sight, hearing, taste, touch, and smell). An overload of this sensory input can cause stress, strain, and imbalance in the mind. Through the mind–body connection, this imbalance can lead to physical disorders.

Ayurveda has various recommendations for stress management and resilience. Increasing Bala and Ojas is important in this regard. Bala refers to strength, including physical strength, mental strength, and strong immunity. Ojas is the end-product of perfect digestion and metabolism at the cellular or tissue level. Ojas maintains and enhances immunity. It also nourishes and sustains the dhatus (tissues) of the body [40]. One of the recommendations for reducing mental stress and increasing Bala and Ojas to increase resilience is Vedic meditation, which utilizes a technique that goes beyond the mind and deep into the inner being, which is a place of peace and bliss. Research has demonstrated the health benefits of this type of meditation [41].

## 5. Conclusions

In conclusion, it is proposed that the genotype and phenotype correspond to Ayurvedic Janma (birth) Prakriti and Deha (body) Prakriti (psychophysiological constitution), respectively. Imbalance or disorder of the Deha Prakriti is known as Vikriti and corresponds to disorders and diseases in the current medical system. It is proposed that mRNA, tRNA, and protein have features and properties that represent the three governing principles of the physiology or Ayurvedic doshas—Vata, Pitta, and Kapha—at the cellular level. There are four major factors that affect the phenotype or Deha Prakriti in a positive or negative way, depending on what one does in one’s life. These four factors are lifestyle and behavior, diet and digestion, stress, and environmental factors. These factors produce changes in the phenotype or Deha Prakriti that affect the expression of the genotype, the Janma (birth) Prakriti, without changing its basic structure. Ayurveda addresses these four major factors of life and thereby affects both the phenotype and genotype in a positive way through the process of epigenetics. Thus, it is proposed that epigenetics is an important mechanism of Ayurveda. This correlation and understanding of the process of healing and health maintenance will improve the understanding and communication between Ayurveda and the current medical system, and lead to better integration of both sciences in the management of optimal health. In addition, research on Ayurvedic modalities affecting gene expression will further increase the correlation and understanding between the current medical system and Ayurveda.
